# Breastmilk transfer of cabotegravir and infant safety outcomes in mothers on cabotegravir for HIV prevention in Lilongwe, Malawi: A protocol for an observational pharmacokinetic study, CABMILK Study

**DOI:** 10.12688/wellcomeopenres.25966.2

**Published:** 2026-06-09

**Authors:** Mphatso S. Kantonya, Clifford G. Banda, Mayamiko D. Kapulula, Steven Munharo, Hellen D. Chavula, Vincent K. Phiri, Diksha Patel, Friday Saidi, Mina Hosseinipour, Fiona Cresswell, Catriona Waitt, Victor Mwapasa

**Affiliations:** 1 Malawi Liverpool-Wellcome Research Programme, Lilongwe, Malawi; 2 The University of North Carolina at Chapel Hill, Chapel Hill, North Carolina, USA; 3 Brighton and Sussex Medical School, Brighton, England, UK; 4 University of Liverpool Department of Women's and Children's Health, Liverpool, England, UK; 5 Kamuzu University of Health Sciences, Blantyre, Malawi

**Keywords:** Pharmacokinetics, PrEP/HIV prevention, Breastfeeding, Maternal-Child Health, Pharmaco-epidemiology, Population Health, Public and Global Health

## Abstract

**Background:**

Long-acting injectable cabotegravir (CAB-LA) is highly effective for HIV pre-exposure prophylaxis (PrEP). However, lactating women have been underrepresented in pharmacokinetic and safety studies despite their elevated risk of HIV acquisition and potential postnatal transmission through breastfeeding. Data on cabotegravir transfer into breast milk and infant exposure remain limited. The Cabotegravir in Mother–Infant Pairs and Lactation Pharmacokinetics (CABMILK) study addresses this evidence gap to inform safe and equitable PrEP delivery within maternal and child health services in Malawi.

**Objectives:**

The primary objective is to quantify cabotegravir transfer into breast milk and estimate infant exposure following maternal use of CAB-LA for PrEP. Secondary objectives are to describe and contextualise infant safety outcomes, including growth, developmental status, and adverse events—among infants whose mothers receive CAB-LA, oral PrEP (tenofovir disoproxil fumarate/emtricitabine), or no PrEP, and to explore maternal factors associated with breast milk drug concentrations.

**Methods:**

CABMILK is an observational pharmacokinetic study nested within two ongoing CAB-LA implementation studies in Malawi. A total of 100 mother–infant pairs will be enrolled: 25 receiving CAB-LA, 25 receiving oral PrEP, and 50 PrEP-naïve. Serial maternal plasma and breast milk samples will be collected to quantify cabotegravir concentrations using validated LC–MS/MS assays and to estimate milk-to-plasma ratios and infant exposure. Infant safety monitoring will include clinical assessments, growth measurements, and developmental evaluation using the Caregiver Reported Early Development Index (CREDI) Short Form over 8 months of follow-up.

**Expected findings:**

This study will provide empirical data on cabotegravir transfer into breast milk, quantify infant exposure, and generate early comparative safety data.

**Conclusions:**

CABMILK will generate essential evidence to guide the safe implementation of CAB-LA among breastfeeding women and inform HIV prevention policy in high-burden settings.

## Plain Language summary

Long-acting injectable cabotegravir (CAB-LA) is a new medicine that can prevent HIV infection and is given as an injection every two months. It is being introduced as an important HIV prevention option for the Malawian population including pregnant and lactating women. However, we still do not know how much of this medicine passes into breast milk when used by breastfeeding mothers, or whether this could affect their babies.

The CABMILK study will help answer these questions. We will follow 100 breastfeeding mothers and their babies. Some mothers will be receiving injectable cabotegravir for HIV prevention, some will be taking daily oral HIV prevention tablets, and some will not be using HIV prevention medicine. We will collect small samples of breast milk and blood from mothers, and tiny blood samples from babies, to measure how much cabotegravir reaches the baby through breast milk. We will also monitor babies’ health, growth, and early development over eight months.

This study will provide important safety information about the use of long-acting cabotegravir during breastfeeding. The findings will help guide doctors, health workers, and policymakers on how to safely offer HIV prevention to breastfeeding women while protecting infant health and assure the community of its safety

## Introduction

Previous HIV Prevention Trials Network studies showed that 2-monthly injectable cabotegravir (CAB) was superior to the daily oral two-drug combination of tenofovir disoproxil fumarate and emtricitabine (TDF/FTC) for HIV prevention in cisgender women. ^1^ Elsewhere, this has also been shown to be true in transgender women and in other groups such as men who have sex with men. ^2, 3^ Due to these promising findings, most countries in HIV-endemic settings are planning to implement CAB use for pre-exposure prophylaxis (PrEP). It is envisaged that CAB would result in improved adherence compared to oral daily PrEP. ^4, 5^ In Malawi, for example, the National HIV programme is developing CAB PrEP guidelines and conducting an implementation study (PathToScale Study), to best understand how to roll out and scale the use of CAB for HIV prevention in all at-risk subpopulations.

However, despite promising evidence on long-acting CAB for PrEP, some subpopulations have not been included in dose-optimisation studies. Breastfeeding women, for example, have been excluded from CAB dose optimisation studies for PrEP, yet are at a high risk of HIV infection for both biological and behavioural reasons. ^6^ In this subgroup, if seroconversion with the associated high viral load occurs at this time, the subsequent transmission risk of HIV to the breastfeeding infant is high. ^7^ Nevertheless, to date, there is a lack of evidence regarding the safety of CAB in mother-infant pairs. ^8^ In particular, the extent of breastmilk transfer of CAB to the infant, its safety profile, and any potential effects on infant growth or development remain unknown. Although CAB is hydrophobic, ^9^ highly protein-bound ^10^ and would unlikely be excreted in breast milk, limited pharmacokinetic data from adult women receiving long-acting CAB formulations indicate detectable but low CAB concentrations in milk. ^11^ A similar pattern of low milk transfer has been described for dolutegravir (DTG), with a reported milk: plasma ratio of approximately 0.03–0.04 from the DolPHIN-1 population PK analysis a structurally similar antiretroviral drug, dolutegravir, has been shown to transfer to infants through breast milk. ^12, 13^ In the study by Dickson et al., some infant DTG exposure was observed postpartum, a finding attributed in part to high transplacental transfer at birth and slower infant elimination, rather than extensive breastfeeding transfer. ^12^ These observations support the expectation that CAB concentrations in breast milk are likely to be low, although direct human CAB lactation data remain limited.

Given the prolonged exposure to LAI CAB, with an apparent half-life of around 40 days, ^9, 14^ there is a need to establish how much CAB transfers to a breastfeeding infant and its associated safety. In the ongoing PathToScale implementation study in Lilongwe and Blantyre, Malawi, the inclusion of diverse subpopulations, such as pregnant women and lactating mothers, will be prioritised to allow the collection of evidence on the safety and acceptability of CAB for PrEP in various key target subgroups. Additionally, an ongoing effectiveness implementation cluster randomised clinical trial is assessing innovative strategies to increase uptake and retention in care for post-partum women who are breastfeeding and initiating long-acting injectable or oral PrEP (LINK Study in Lilongwe, Malawi). These two implementation studies provide a unique platform to nest a cohort study to understand the breastmilk transfer of CAB to infants and its subsequent safety in these young children. Through the cabotegravir in mother-infant pairs and lactation pharmacokinetics study (CABMILK Study), we aim to address this knowledge gap by determining the extent to which CAB is transferred to the infant through breastmilk, and explore whether CAB breastmilk concentrations, if any, are high enough to cause adverse effects in the infant, and affect growth or neurocognitive development.

## Main and specific objectives of the CABMILK study

The broad objective of the CABMILK Study is:

• To characterise cabotegravir transfer into breastmilk and the resulting infant exposure among breastfeeding women receiving long-acting injectable cabotegravir for HIV pre-exposure prophylaxis, and to describe, in a preliminary, non-inferential manner, the infant safety, growth, and developmental patterns in this group relative to infants whose mothers receive oral PrEP (TDF/FTC) or no PrEP.

The specific aims of the study are:

• To evaluate the extent of cabotegravir transfer into breast milk and determine its pharmacokinetics in infants who are breastfed by mothers receiving long-acting injectable cabotegravir for HIV PrEP.
• To describe the pattern of infant safety, growth, and developmental outcomes over eight months of follow-up among infants exposed to cabotegravir through breastmilk, those exposed to oral TDF/FTC through breastmilk, and PrEP-naïve infants, with effect sizes and 95% confidence intervals reported without formal between-group hypothesis testing

## Methodology

### Study design and population

**Figure 1.**
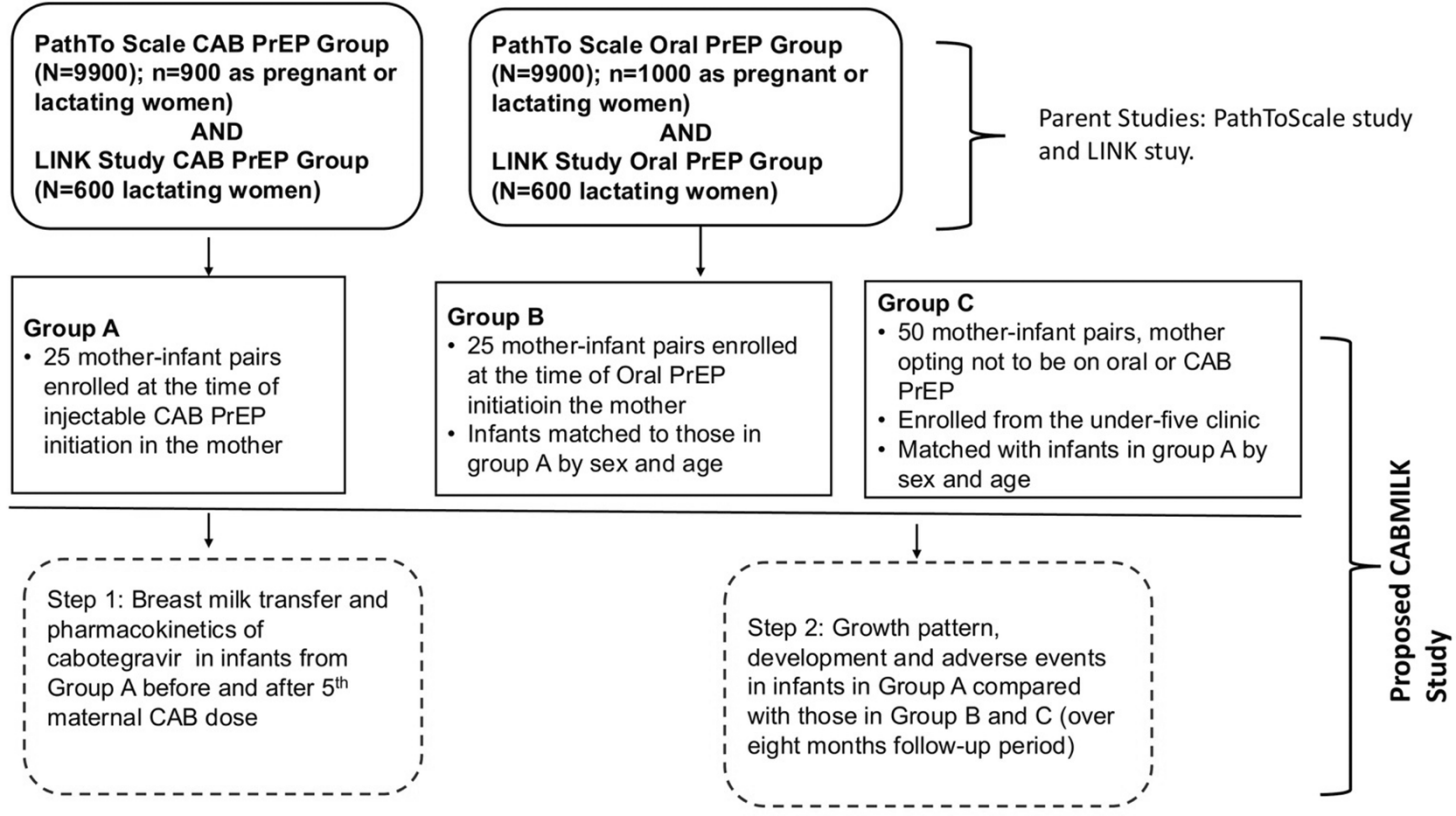
Study design and follow-up schedule for the CABMILK study. Schematic overview of the CABMILK observational pharmacokinetic cohort study. Lactating mother–infant pairs are enrolled into one of three groups based on their HIV pre-exposure prophylaxis (PrEP) choice: injectable cabotegravir (CAB-LA), oral PrEP (tenofovir disoproxil fumarate/emtricitabine), or no PrEP. The diagram illustrates the expected number per group, timing of PK samples in group A, alongside clinical follow-up visits for safety, growth, and developmental assessments over the eight months follow up.

To address the outlined study aims, an observational pharmacokinetic cohort study ( Figure 1), will be conducted in two steps. In the first step, lactating mothers under the PathToScale or LINK studies who choose CABLA for PrEP will be targeted for enrolment in order to profile the extent of breastmilk CAB transfer to the infant following maternal intramuscular CAB administration for PrEP. In the second step, an assessment of the safety outcomes (growth, neuro-cognitive development patterns and incidence of adverse events) in infants exposed to breastmilk CAB compared with those exposed to oral PrEP or PrEP-naïve infants will be made. The CABMILK Study will be nested within two ongoing CAB PrEP studies in Malawi, the PathToScale Study and the LINK Study ( Figure 1). The PathToScale Study aims to assess CAB implementation readiness and outcomes (feasibility, acceptability, appropriateness, adaptation, and fidelity) according to the preliminary national CAB PrEP guidelines in Malawi (currently under development). PathToScale will recruit 9,900 participants into the CAB group and 13,000 participants into the standard of care (oral daily PrEP of the TDF/FTC combination) between January 2024 and December 2026. The LINK Study is an effectiveness implementation cluster randomised clinical trial that is assessing innovative strategies, such as the alignment of PrEP services to routine under-five clinic visits, to increase uptake and retain in-care postpartum women who are breastfeeding and initiating long-acting injectable or oral PrEP. ^15^ LINK will randomise 12 clinics in Lilongwe District into two clusters: one with improved innovative strategies for increasing uptake of CAB and oral PrEP and a second cluster of standard care. Combined, both clusters are expected to recruit at least 600 breastfeeding women between 2024 and 2027 (150 at the standard of care sites and 450 at the intervention sites over three years) and follow them up.

In the CABMILK Study, three groups of mother-infant pairs will be included as follows: i) 25 mother-infant pairs from a group of lactating mothers who opt to receive long-acting CAB injectable ii) 25 mother-infant pairs from a group of lactating mothers who opt to receive standard oral PrEP comprising tenofovir disoproxil fumarate and emtricitabine, and iii) 50 mother-infant pairs from a group of lactating mothers who chose not to be on oral or injectable PrEP (PrEP unexposed group) ( Figure 1). While this study leverages data collection platforms established by PathToScale and LINK for efficiency, the CABMILK Study independently focuses on evaluating cabotegravir transfer into breast milk and its association with infant safety. The research questions and analyses have been designed to address gaps not explored in the parent studies, ensuring originality and relevance to the local context.

### Study place and setting

The study will be conducted in at least two health facilities in Lilongwe district (Area 18 Health Centre and Kawale Health Centre under the clinical research platform of the University of North Carolina (UNC)-Lilongwe project) where long-acting CAB and oral PrEP are being offered as part of the PathToScale and LINK studies. These have been identified in consultation with the Health Offices in both districts and key implementing partners. The choice of a site has also been made based on the availability of maternity care services at the health facilities. All sites will be expected to contribute towards this study’s total sample size (n=100), depending on the levels of participant recruitment in the parent studies.

### Inclusion and exclusion *criteria*

*The inclusion criteria of the study are:*

Eligible participants will be breastfeeding mother–infant dyads recruited from health facilities participating in the PathToScale and LINK implementation studies. Inclusion criteria are designed to identify HIV-negative breastfeeding mothers with anticipated or documented exposure to oral or long-acting injectable cabotegravir for HIV pre-exposure prophylaxis (PrEP), while ensuring the feasibility of pharmacokinetic sampling and infant safety follow-up.

Specifically, eligible mothers will be HIV negative, aged 15 years or older and are either: (i) intending receive oral or injectable cabotegravir PrEP under government facilities which maybe PathToScale or LINK studies implementation sites, or (ii) HIV-negative breastfeeding mothers attending postnatal or under-five clinics at participating facilities. Mothers will be expected to be willing and able to comply with all study procedures as described in the Participant Information Leaflet (PIL) and to provide written informed consent for themselves and their infants or assent for participants less than 18 years of age. It will be expected that infants remain within the infant age range at the time of planned mother–infant pharmacokinetic sampling to allow adequate assessment of cabotegravir exposure and safety outcomes. As such enrolment for eligible mother-infant pairs shall occurs from the first week of birth to less than 5 months.

*The exclusion criteria of the study are:*

Exclusion criteria will be applied primarily to protect participant safety and preserve the interpretability of pharmacokinetic measurements. Mother–infant dyads will be excluded if either the mother or infant is receiving concomitant medications known to significantly alter cabotegravir exposure, including rifamycin--based tuberculosis prevention or treatment. Enrolment will be restricted to infants with a birthweight ≥1.5 kg; extremely preterm and clinically unstable neonates are therefore excluded, but otherwise well preterm infants are eligible in order to preserve generalisability to the real-world population receiving CAB-LA under the parent studies. Gestational age at birth will be recorded at enrolment and used as a covariate in analyses. Dyads will also be excluded if the infant has a documented congenital anomaly expected to affect drug disposition or survival. Severe anaemia, defined as haemoglobin concentration < 5 g/dL, will constitute an exclusion criterion. In addition, participants will be excluded if they are found to be concurrently enrolled in another interventional clinical trial, with the exception of the PathToScale or LINK implementation studies.

### Data collection techniques and tools for the CABMILK study

**Figure 2.**
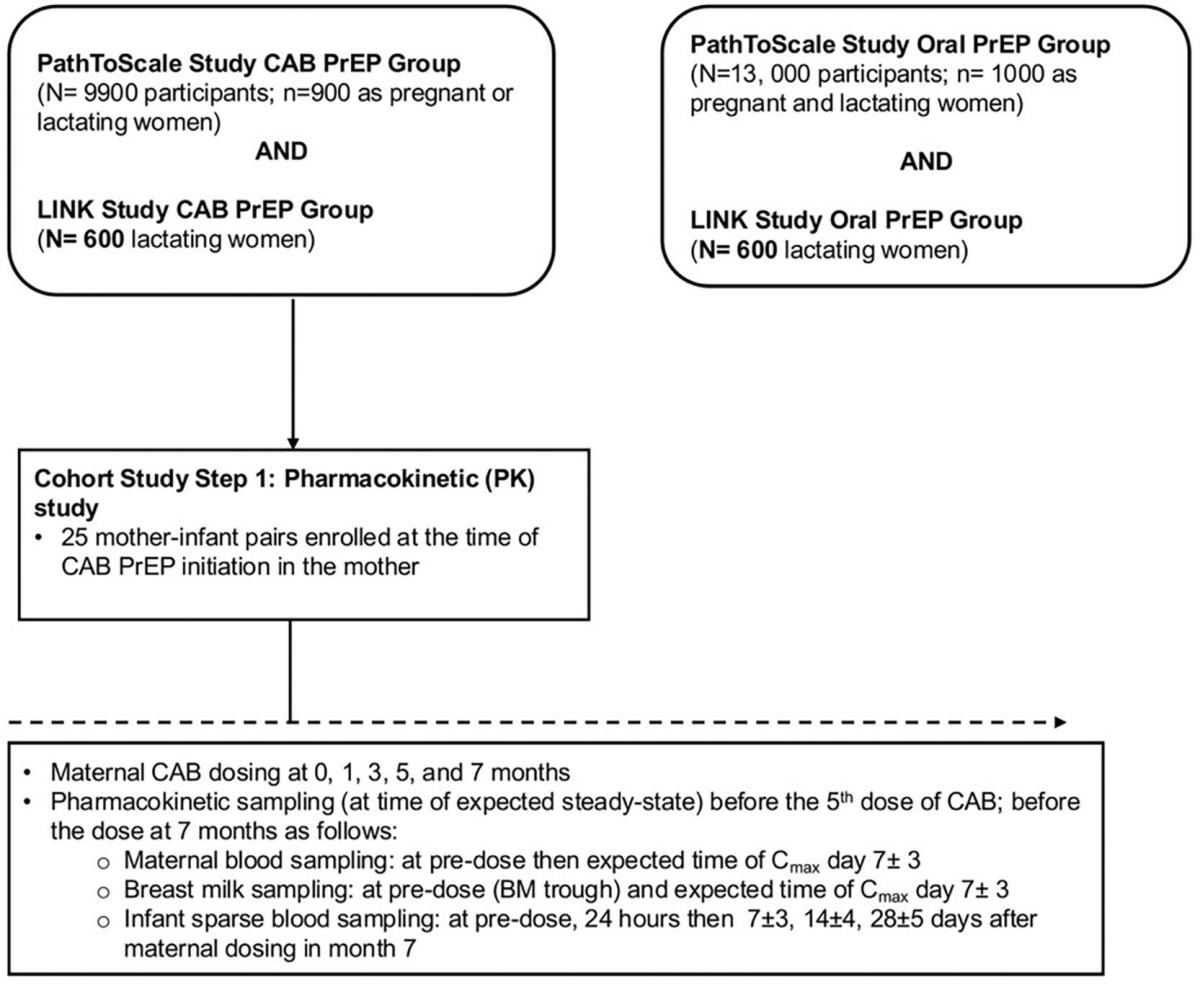
Pharmacokinetic sampling schedule for the CABMILK study. Timeline of maternal plasma, breastmilk, and infant dried blood spot (DBS) sample collection used to characterize cabotegravir pharmacokinetics and infant exposure during breastfeeding. The sparse PK sampling visit occurs at month 7 after enrolment, corresponding to the fifth CAB-LA injection, after participants have received four prior injections. This schedule enables estimation of maternal drug concentrations, milk-to-plasma ratios, and infant exposure metrics.

Procedures in the CABMILK study are outlined in Figure 2 and Table 1. Data will be collected using case-report forms (CRF) that were developed using open data kit (ODK) platform.

**Table 1.** Schedule of study procedures and sample collection in the CABMILK study.

Visit description											
Visit number	1	2	3	4	5	6	7	8	9	10	Unscheduled
Visit month	0	1	3	5	7	7	7	7	7	8	Any
Visit study time	Pre- dose	Pre- dose	Pre- dose	Pre- dose	Pre-dose (0 hr)	24 hr	Day 7±3	Day 14±3	Day 28±5	Study exit	Sick visit
Screening and enrolment											
Informed consent discussion	x										
Medical and drug history	x										x
Revision of eligibility criteria	x										
ID number assignment	x										
Capillary haemoglobin measurement	x				x						
Group A: Mother-infant pairs on cabotegravir PrEP											
General physical examination	x	x	x	x	x	x	x	x	x	x	x
Recording of current and concomitant medication	x	x	x	x	x	x	x	x	x	x	x
Anthropometric measurements to assess growth ^#^	x	x	x	x	x					x	
Cognitive and neuro-assessment using a validated tool such as MDAT or equivalent	x	x	x	x	x					x	
Adverse events assessment	x	x	x	x	x	x	x	x	x	x	
Maternal venous blood PK sample					x		x				
Mandated breastmilk feeding or record of latest time of feeding*							x				
Maternal breastmilk PK sample					x		x				
Infant capillary blood PK sample					x	x	x	x	x		
Infant feeding patterns and record of any food supplementation	x	x	x	x	x	x	x	x	x	x	x
Groups B & C: Mother-infant pairs in oral PrEP group and PrEP naïve											
General physical examination	x	x	x	x	x					x	x
Recording of current and concomitant medication	x	x	x	x	x					x	x
Anthropometric measurements to assess growth ^#^	x	x	x	x	x					x	
Cognitive and neuro-assessment	x	x	x	x	x					x	
Adverse events assessment	x	x	x	x	x					x	

This table outlines the timing of study visits and procedures for enrolled mother–infant pairs, including maternal clinical assessments, breastmilk and plasma sample collection, infant dried blood spot (DBS) sampling, and safety, growth, and developmental evaluations. Procedures are aligned with maternal cabotegravir dosing schedules and routine follow-up visits.

### Cabotegravir injectable and oral PrEP administration

Long-acting CAB in the parent PathToScale and LINK implementation studies will be administered as a 600 mg intramuscular injection in the ventrogluteal region by a trained provider certified by the MoH on the same day that a client is determined to be eligible. This will be followed by a second injection 1 month after the first and subsequent injections every 2 months until the final injection at eight months. Oral PrEP will be administered as a daily single tablet of 300 mg tenofovir disoproxil fumarate/200 mg emtricitabine. Participants in this group will be followed up until eight months in our study.

### Step 1- CABMILK study- Cabotegravir maternal, breastmilk and infant pharmacokinetic study

We hypothesise that cabotegravir administered as long-acting PrEP in lactating mothers may be detectable in breast milk and infant plasma, but that the magnitude of infant exposure and its clinical significance remain uncertain. The study is designed to quantify breast milk transfer, estimate infant systemic exposure, and evaluate infant safety. If infant exposure occurs at very low, subtherapeutic concentrations, this could have theoretical implications for resistance in the event of HIV acquisition during breastfeeding; however, it is also possible that transfer is minimal and clinically reassuring and safe. CAB would transfer.

*Cabotegravir pharmacokinetic sampling: maternal plasma, breastmilk, and infant plasma*

25 lactating mothers initiating CAB PrEP within the PathToScale Study or LINK Study will be approached for participation in the study. Since CAB has a half-life of 40 days ^9, 14^ it would be expected to attain a steady state in the mother at least after 4 half-lives ^16^ (i.e. in the case of CAB, at least 160 days) from the first initial dose. To ensure that we conduct pharmacokinetic sampling of CAB at steady state, we will collect samples for CAB concentrations before the 5th CAB maternal dose (i.e. before the maternal dose at 7 months postpartum).

In mothers, 5 mL of venous blood will be collected using calibrated EDTA tubes. It will be separated in the laboratory to collect plasma and frozen in at least two aliquots at -80 degrees Celsius in readiness for a validated liquid chromatography–tandem mass spectrometry (LC–MS/MS) assay. Breastmilk CAB samples will be paired and collected by manual expression at pre-dose and at the expected time of maternal peak (Cmax), 7 days (±3) post-5th CAB dose. No more than 5 mL of breast milk will be collected in a clear container at each sampling time point. Thereafter, the sample will be frozen in at least two aliquots and stored at -80 degrees Celsius in readiness for cabotegravir assay. Therefore, each mother will contribute two breastmilk and blood samples.

To characterise and profile infant CAB exposure from breastmilk, 200 μL of infant capillary blood will be collected through a finger/heel prick at the expected time of trough- (sample collected before dosing in the mother) and peak (expected Cmax) concentrations at 7 days (±3) ^17^ post-dose. The latter will be achieved by aiming to mandate a feed at the expected maternal peak and then sampling 1 hour later or, where mandated feeding is not possible, collecting information on the time of last infant breastfeeding. We will further collect infant capillary blood samples at 24 hours then 7 (±3), 14 (±4) and 28 (±5) days post maternal CAB dosing, resulting in five infant sampling occasions ( Figure 2). The collected blood sample will be applied on two dry blood spot (DBS) filter papers (Whatman 3MM) and left to dry. The DBS filter papers for each participant will be stored, with desiccant, in sealed zipper plastic bags to ensure that they do not pose any potential biological hazards. They will be stored at room temperature at a collaborating institution, the University of North Carolina (UNC)’s laboratory before shipment to assay cabotegravir concentrations. The remaining samples will be archived at Malawi-Liverpool-Wellcome Programme (MLW)’s laboratory in Blantyre.

Ideally, venous plasma samples, as opposed to capillary samples, should also be collected in infants (as in the case of the mother). However, to limit the amount of infant blood sampling volume, a capillary blood sampling matrix has been opted for in the infants.

*Duration of sample storage*

All samples (breast milk and blood) will be collected in duplicate; one backup at the Malawi-Liverpool Wellcome Programme (MLW) laboratory and the other shipped for cabotegravir assay. The maximum period of sample storage at MLW (backup samples) will be five years.

*Assaying cabotegravir concentrations*

Breast milk and blood samples will be shipped to the University of Cape Town’s Division of Clinical Pharmacology Laboratory or the Infectious Diseases Institute Laboratory in Uganda for quantification of cabotegravir concentrations. Samples will be analysed using a validated liquid chromatography–tandem mass spectrometry (LC–MS/MS) assay capable of detecting very low concentrations of cabotegravir in plasma, breast milk, and dried blood spot matrices. The assay has been validated in accordance with international bioanalytical method validation guidelines, including assessment of accuracy, precision, recovery, matrix effects, and analyte stability. The lower limit of quantification (LLOQ) is expected to be in the low ng/mL range, sufficient to detect concentrations anticipated in infant samples. External quality control samples and calibration standards will be included in each analytical run.

Shipment of samples outside Malawi is required because this highly sensitive LC–MS/MS method for cabotegravir is not currently available locally.

*Information on other factors that could impact CAB breastmilk exposure*

Two classes of variable are recognised as potentially affecting cabotegravir concentrations in breastmilk and the resulting infant exposure: (a) infant feeding pattern, and (b) maternal nutritional status. Infant feeding will be captured at every visit using standard feeding indicators (exclusive vs mixed) and the timing and frequency of feeds relative to sampling and will be entered into pharmacokinetic and safety analyses as a time-varying covariate. Maternal pre-injection weight will be measured at baseline and at each dosing visit and evaluated as covariates on cabotegravir apparent clearance and on the milk-to-plasma ratio in the population PK model. We acknowledge as a limitation that the study does not directly measure milk fat fraction or detailed maternal dietary intake, and therefore cannot fully resolve any indirect effect of maternal diet on infant cabotegravir exposure.

### Step 2- Cabotegravir safety, growth pattern and development study

We hypothesise that there will be no difference in the incidence of adverse events or illnesses, growth, and development between infants exposed to breast milk CAB and those exposed to breast milk oral PrEP. 25 mother-infant pairs from Step 1 (mothers receiving CAB for PrEP), 25 other mother-infant pairs from the oral PrEP group of the PathToScale and LINK studies and 50 mother-infant pairs naïve of PrEP, matched to those in the CAB PrEP group, will be followed up for eight months from the time of maternal PrEP initiation in the PrEP groups or a similar time in the PrEP naïve group. The PathToScale and LINK studies are following up mothers in the CAB group from the initiation visit, 1 month later and, thereafter, every 2 months. In our study we will follow a similar schedule for all three groups with a final exit visit scheduled at eight months post initial CAB dose. This will result in 10 scheduled visits in the CAB group and six in the oral PrEP and no PrEP groups. The CAB LA PrEP group will have four additional visits for PK sampling at month 7. In the present CABMILK Study, we will utilise all six planned visits to assess the incidence of reported adverse events and infant growth by measuring anthropometric indices (weight-for-height and height-for-age) as well as head circumference-for-age. The same visit schedule and safety assessments will be applied to the oral PrEP and PrEP-naïve groups. Infant growth and neurodevelopment will be longitudinally assessed at all six study visits using the Caregiver Reported Early Development Instrument (CREDI).

### CREDI tool

Child development will be assessed using the Caregiver Reported Early Development Index (CREDI) – Short Form, a caregiver-reported instrument designed for children from birth to 36 months. Trained study staff will guide caregivers through the structured questions, and the CREDI Short Form responses will be scored using the official CREDI scoring algorithms and reference parameters to generate age-standardised developmental status scores. The tool captures developmental milestones across key domains including motor, language, cognitive and socio-emotional development and yields a summary score reflecting overall developmental status. The tool has been validated for population-level measurement in diverse cultural settings and has demonstrated good internal consistency and concurrent validity with more intensive clinician-administered instruments such as the Bayley Scales of Infant and Toddler Development. ^18^

In this study, developmental outcomes will be analysed as continuous summary scores and compared across exposure groups using longitudinal regression methods. The study prioritises comparability and feasibility over clinical diagnosis, and findings related to child development will therefore be interpreted as indicators of group-level developmental patterns rather than diagnostic outcomes for individual infants. The use of CREDI allows for standardized developmental assessment across visits and study groups, while remaining practical and culturally appropriate for use in routine clinical and implementation research settings. ^19^

### Missed visits

All dosing visits for injectable and oral PrEP are aligned with those of the parent PathToScale and LINK implementation studies. As the CABMILK study is leveraging the platform of the implementation of PathToScale and LINK studies, missed dosing or follow-up visits are inevitable. Nevertheless, any such missed dosing or follow-up visits, including sample collection visits, will be conducted as soon as it is possible to do so. This will be documented appropriately in the participant’s file.

Retention and evaluable pairs: The primary pharmacokinetic analysis will be conducted on mother–infant pairs who contribute matched maternal plasma, breastmilk, and at least one infant dried blood spot sample within the month-7 window. Losses before the month-7 sampling visit, missed CAB-LA doses, and out-of-window sampling will be documented and reported as implementation outcomes. Baseline characteristics of mothers who do and do not reach the evaluable PK sampling visit will be compared descriptively. Retention is supported through appointment reminders, alignment of CABMILK visits with routine postnatal and under-five clinic contacts, travel reimbursement, and, where acceptable to the mother, a home visit to complete a missed sampling occasion. Recruitment will continue until the target of 25 evaluable pairs in the CAB-LA group has been met bearing in mind study timelines.

### Sample size determination and power calculation

Sample size calculation has been primarily based on step 1 of the CABMILK study. Step 2 leverages on the previous step. In Step 1 (Cabotegravir maternal, breastmilk and infant pharmacokinetic study), previous data on 600 mg intramuscular CAB in adults yielded an area under the concentration-time curve (AUC0-∞, μg*h/mL, [range]) of 4172 [2839–6597] with a coefficient of variation of 24%. ^17^ Assuming a similar coefficient of variation, the inclusion of 25 breastfeeding infants will provide >95% precision to estimate overall infant CAB exposure.

Step 2 (Prospective observational cohort follow-up of mother–infant pairs): Detecting rare safety outcomes or subtle neurodevelopmental effects related to cabotegravir exposure would require substantially larger and longer-term studies. Accordingly, Step 2 is designed as a prospective observational cohort to describe infant exposure and characterise safety signals rather than to support formal inferential comparisons. Safety outcomes will include reported adverse events, growth patterns, and changes in developmental status over time.

We plan to enrol 100 mother–infant pairs (25 CAB-LA, 25 oral TDF/FTC PrEP, and 50 PrEP-naïve). This sample size is intended to provide a reasonable descriptive profile of safety and developmental outcomes across exposure groups. The larger PrEP-naïve group will support more stable estimation of background patterns against which findings in the exposed groups can be contextualised.

Child development will be assessed using the CREDI Short Form, a caregiver-reported tool that provides a summary developmental status score suitable for population-level description rather than clinical diagnosis. Assessments over 8 months of follow-up will allow characterisation of short-term developmental trajectories, while recognising that longer-term outcomes cannot be determined within this study.

Comparative analyses will be exploratory. Longitudinal growth indices will be analysed using linear mixed-effects models (or generalized estimating equations) to account for repeated measures, and developmental status (continuous CREDI summary score) will be compared across groups using adjusted regression models. Because exposure groups are not randomised, analyses will be interpreted as observational associations and adjusted for prespecified maternal and infant confounders. Results will emphasise effect sizes and confidence intervals. We acknowledge that the study is not powered to detect subtle differences, rare events, or long-term neurodevelopmental outcomes, and findings will therefore be interpreted cautiously and used to inform the design of future larger studies.

### Data analysis

Data will be analysed using either STATA or R. Table 2 outlines the planned data analyses stratified by the specific objectives of the study. Infant age at every sampling occasion and at every safety and developmental assessment will be recorded to the day. Both postnatal age (PNA) and postmenstrual age (PMA = gestational age at birth + PNA) will be derived for each timepoint and treated as time-varying variables. The population pharmacokinetic model (NONMEM/Monolix) will incorporate, as fixed structural features, allometric size scaling (exponent 0.75 on apparent clearance, 1 on volume of distribution) and a sigmoidal maturation function on apparent clearance, with PMA50 and the Hill coefficient set to published values for UGT1A1 ontogeny rather than estimated from this small cohort. Residual covariate effects of PNA, weight, sex, and feeding pattern will then be tested empirically. To probe the robustness of the maturation assumption, we will conduct a pre-specified sensitivity analysis re-fitting the model with at least one alternative published UGT1A1 ontogeny parameter set (1) and report the resulting impact on individual exposure estimates. Relative infant dose (RID) will be reported overall and by infant-age stratum (<9 months and 9–<12 months). Comparative growth and developmental analyses will use WHO z-scores and age-adjusted CREDI scores; longitudinal mixed-effects models will include infant age at measurement as a continuous covariate. Because exposure groups are self-selected, all comparative analyses are observational and exploratory. Primary safety results will be descriptive: counts, proportions with exact 95% confidence intervals, incidence rates, and Kaplan–Meier curves, without group-comparison p-values. Longitudinal growth and CREDI outcomes will be analysed with mixed-effects models adjusting for a minimal pre-specified set of covariates: infant age at measurement, infant sex, and site. Formal between-group inference for safety is deferred to other larger PK studies. Where feasible, we will perform sensitivity analyses to reduce imbalance, recognising limitations due to our sample size.

**Table 2.** Planned data analyses aligned with study objectives in the CABMILK study.

Objective	Data analysis	Outcome
To profile the extent of CAB transfer in breast milk and determine its pharmacokinetics in infants whose mothers receive long-acting injectable cabotegravir for HIV preexposure prophylaxis (PrEP).	We will use non-linear mixed effects models to determine the compartmental pharmacokinetics of CAB from maternal plasma to breast milk to infant plasma. We will first calculate the milk: plasma ratio to determine the average milk CAB concentration compared with the average maternal plasma concentration. Second, we will utilise individual predicted infant CAB concentrations to calculate infant overall exposure (AUC0-∞) and estimate CAB infant half-life as well as elimination rate constant. Third, we will estimate the CAB dose ingested by each infant, through breast milk, relative to the maternal dose; the relative infant dose (RID). ^25^ This RID will allow us to determine the extent of CAB breast milk exposure that would result in attained concentrations in the infant’s blood that are above or below the protein-adjusted IC90 of 0.166 μg/mL. ^26^	CAB transfer to the infant through breast milk, and profile the attained infant concentrations.
To determine the association of breast milk CAB exposure and infant safety outcomes (growth pattern, neuro-cognitive development, and incidence of adverse events).	Firstly, mean anthropometric indices (weight-for-height, height-for-age, head circumference-for-age) will be compared across three groups using independent t-tests. Secondly, adverse events (overall morbidity) by the end of follow-up will be compared across the three groups using Chi-squared tests. Age-standardised CREDI summary scores (z-scores) analysed as continuous outcomes, reported as adjusted mean differences with 95% confidence intervalsUsing linear regression, we will assess whether breast milk CAB concentrations predict growth patterns and morbidity, adjusting for infant age, sex socio-economic status, birth weight and gestation age at birth. Random effects models will account for intra- and interindividual variability in repeated measurements. Exploring this analysis may inform algorithms for monitoring CAB safety signals in infants. These algorithms could be validated in larger studies and incorporated into routine practice for early detection of CAB-related risks in infants.	Impact of CAB breast milk exposure on infant growth and neurocognitive development in comparison to the standard daily oral PrEP.

This table outlines each primary and secondary study objective alongside the corresponding outcomes, exposure measures, and planned statistical or pharmacokinetic analyses. It summarises how each objective will be addressed analytically, including comparisons across maternal PrEP exposure groups and evaluation of infant pharmacokinetic, safety, growth, and developmental outcomes.

## Ethical considerations

The CABMILK study protocol, participant information sheet, and informed consent documents have been reviewed and approved by the National Health Sciences Research Committee (NHSRC) of Malawi (approval reference 24/10/4527), with institutional ethical oversight from the Malawi-Liverpool-Wellcome Research Programme Clinical Research Support Unit (CRSU). The study will be conducted in accordance with the Declaration of Helsinki (2013) and ICH Good Clinical Practice (E6 R2, and R3 as applicable). Any protocol amendments will be submitted to the NHSRC before implementation, and serious adverse events will be reported within the regulatory timelines.

### The informed consent process

During consenting, the literacy level of the mother will be assessed by first asking her whether she would be willing to read and explain a brief statement in the consent form. To minimise undue bias of socioeconomic status within the study population, mothers who are unable to read or write will still be considered for participation in the study as long as the mother understands the participant information sheet (PIS) and there is an impartial witness during the informed consent process to ensure that the mother is provided with correct and complete information. The mother will be asked if they are comfortable with this arrangement. The impartial witness will be someone who is not directly involved with the study e.g. a companion of the mother or another health worker from the government health facility who is literate. Informed consent will be obtained using the following process:

• The potential participant (mother) will be invited into a private space to discuss the consent form with a study nurse in their local language.
• The study nurse or study physician or principal site investigator will review the form with the potential participant and assess understanding of the study by asking the participant to repeat the main message of each part of the consent form. Every effort will be made to explain the process and procedures of the study in a language that the participant could understand.
• In cases where a potential participant displays a clear lack of understanding of the aim of the study, procedures, time commitment, risks and benefits, assessed through question-and-answer sessions, and after several attempts to clarify them, the study personnel will be allowed to make a judgement not to continue with the consent process. To avoid causing embarrassment to the mother, the study personnel will be required to decide with the mother by highlighting that on initial assessment, it would appear that the study may not be suitable for her or her child.
• The study personnel will then provide time for the mother to ask any questions that she may have and will make a record of these questions and the responses that are given. The mother will be given the PIS to take home to read or discuss the information with any other person (if they wish), including members of their family, friends or a healthcare worker that they see routinely. They will be requested to allow the study team to contact them the following day, either through a mobile phone or home visit, for the participant to ask any further questions and to communicate their decision.
• If the mother agrees to participate in the study, she will be asked to sign the consent form in the presence of the study personnel, and an impartial witness if the participant is illiterate. As consenting is a continued process, study nurses will continue to confirm, verbally, the mother’s willingness to continue participating in the study at each study visit.

### Withholding information

No information will be withheld from the study participants. Any relevant new findings will be shared with the National Health Sciences Research Committee (NHSRC) and study participants, with re-consenting as appropriate.

### Risk of study participation: venous sampling, breastmilk sampling, finger/heel pricks and mitigation of potential injury

Participants will undergo repeated needle and finger/heel pricks in this study, than they normally would, but in line with standard hospital procedures in relation to blood sample collection. The risks of these procedures include pain, transient bleeding, and soft tissue infection. A small bruise or mild pain at the site from where the blood is taken may develop. To mitigate this risk, only well-trained study staff will be hired for the project. Additionally, only new disposable needles and lancets will be used for the blood collection procedures, which will be safely discarded immediately after use. Furthermore, breastmilk manual expression, as part of sampling, may be associated with some minimal risk of discomfort in mothers and the need for privacy. To mitigate this, all mothers will be supported by trained study team members to manually express breastmilk comfortably. In addition, all sampling occasions will be conducted in a private room/space to ensure comfort and maintain privacy.

### Recommended infant blood sample volume

Infant blood will be collected using calibrated micro-collection EDTA tubes, and each infant will contribute five blood samples ( Figure 2). The total infant blood volume will not exceed 5 mL for all blood sampling time points. This volume is safe for an infant as per NIHR guidelines that state that that blood volume limits for sampling, per individual (including study-related blood loss and any losses in the manoeuvre), should not exceed 3% of the total blood volume in a period of four weeks, and should not exceed 1% at any single time. For a 5 kg infant, this equates to 4 mL per sample/at a single sampling time point or 12 mL over 4 weeks. ^20^ A total infant blood volume of 5 mL for all blood sampling time points in the CABMILK study is therefore safe. In all study procedures, loss of blood or excess blood sample collection will be minimized, during sampling, by using calibrated capillary and venous collection tubes.

### Benefits of participation in the study

The direct benefit of participation in the CABMILK study is that infants would be closely monitored, and this would be particularly valuable in this sub-population, which is at higher risk of general childhood illnesses. The indirect benefit is that the study is envisaged to provide evidence on the safety of cabotegravir breast milk exposure during childhood. Although the burden of research to inform the safety of breastmilk exposure to cabotegravir is being borne by the participants in this study, the evidence will likely inform policy and practice and thereby be applied to a wider population.

### Privacy and confidentiality

During this study, medical history and physical examination will be performed at baseline and regular intervals. All the materials collected are for research purposes only, and data will be kept in strict confidence. No information will be given to anyone without permission from the subject. The consent form includes the informed consent statement which guarantees confidentiality. Confidentiality will be ensured using identification codes assigned to the study participants. All data, whether generated in the laboratory or at the bedside will be identified using an identification code unique to the subject. The data managers at MLW will combine the baseline, pharmacokinetic data and follow-up data into a final data set which will be password protected. Handling and processing of data will be according to data protection legislation in Malawi.

### Duration of sample and data storage

All samples (breastmilk and blood) will be collected in duplicates; one backup at the Malawi-Liverpool Wellcome Programme (MLW) /UNC Project laboratory and the other shipped for cabotegravir assay. The maximum period of sample (backup sample) and data storage at MLW will be five years following the completion and publication of the study findings.

### Dissemination and data sharing plan

Community engagement in CABMILK was initiated at study set-up and continue throughout implementation. Sensitisation meetings were held with hospital staff, and later with community leaders, and residents in the catchment areas of the participating health centres. Ongoing engagement will take place through listening sessions with enrolled mothers and caregivers in Chichewa at the under-five and postnatal clinics, and through community-engagement activities broadly organised by the institution’s Community Engagement Department aimed at increasing visibility in Lilongwe, where its research activities are not yet well established. From these activities, we aim to enhance enrolment and retention in the CABMILK study. These CABMILK-specific activities are distinct from, but co-delivered with, the broader community-engagement architecture of the parent PathToScale and LINK studies. These activities ensure that the study remains transparent, culturally grounded, and responsive to community needs.

Community engagement forms the backbone of the CABMILK study. Initial sensitisation meetings will be held with hospital staff, community leaders, and residents to introduce the study and address early questions. Ongoing engagement will continue through outreach at government-supported under-five and postnatal clinics, small-group discussions in local languages, and a dedicated community radio program developed with health educators. These activities ensure that the study remains transparent, culturally grounded, and responsive to community needs.

Scientific dissemination will focus on contributing to the global evidence base for HIV prevention in lactating women. The findings will be submitted to reputable peer-reviewed journals and shared at major international conferences, including the IAS. Additional methodological outputs, such as breast milk pharmacokinetic analyses and safety signal detection models, will be published as preprints and made accessible through open science platforms.

To support policy and programmatic decision-making, tailored briefs and technical summaries will be provided to the Malawi Ministry of Health, the NHSRC, and other national stakeholders. The results will be presented in multisectoral learning sessions to inform national CAB-LA rollout and strengthen maternal–child health pharmacovigilance. A key output will be early algorithms for detecting potential safety concerns in infants exposed to cabotegravir through breast milk, with the aim of integrating these into routine monitoring systems.

Public dissemination will prioritise accessibility and clarity. Lay summaries, infographics, and materials in English and Chichewa will be shared through health facility posters and digital platforms such as social media and WhatsApp. Engagement with community advisory boards and mother support groups will ensure that the findings are understood and can inform community dialogue.

Data sharing will follow open science principles, while protecting participants’ confidentiality. Aggregated datasets, breastmilk pharmacokinetic summaries, and analysis codes will be shared on Wellcome Open Research and an MLW-managed repository. De-identified individual-level data will be available upon reasonable request under a governed access model. Protocols, analysis plans, and metadata will be openly accessible to support reproducibility and wider scientific use.

### Study status

The study started recruitment in September 2025, and enrolment is currently ongoing.

## Discussion

Long-acting injectable cabotegravir (CAB-LA) has recently been recommended by the WHO as an additional HIV prevention option for individuals at a substantial risk of infection, supported by strong efficacy and safety data from randomised trials. ^21^ Despite this progress, WHO highlights a critical evidence gap regarding the safety, pharmacokinetics, and programmatic implications of CAB-LA use during pregnancy and breastfeeding (two periods associated with heightened HIV susceptibility and the potential risk of vertical transmission. ^7^ In response, Malawi endorsed the introduction of the CAB-LA through a national implementation science platform that explicitly included lactating women as an important subpopulation requiring targeted evidence. ^22, 23^

To our knowledge, the CABMILK study is the first to leverage this national CAB-LA rollout platform to systematically characterise cabotegravir exposure in lactating mothers initiating injectable postpartum PrEP. By measuring cabotegravir concentrations in both maternal plasma and breast milk after achieving a steady state (i.e. after five injections, ^16^ this study will quantify the extent and pattern of transfer into breast milk across the dosing interval). Subsequent measurement of cabotegravir concentrations in breastfed infants will allow us to determine the degree of infant exposure and the pharmacokinetic relationship between the maternal blood, milk, and infant compartments.

This work addresses a fundamental pharmacological question: whether cabotegravir, given its hydrophobicity ^9^ and high protein binding, ^10^ exhibits minimal excretion into breast milk, as predicted. Or whether measurable transfer occurs, similar to reports for other integrase inhibitors such as dolutegravir. ^12, 13^ If cabotegravir is detectable in infant plasma, we will assess whether concentrations approach thresholds associated with antiviral activity or selection pressure, thereby revealing the potential risk of resistance emergence.

In addition to pharmacokinetic outcomes, CABMILK provides a unique opportunity to compare infant growth, morbidity, and early developmental milestones among infants in three exposure groups: CAB-LA, oral PrEP (TDF/FTC), and PrEP-naïve groups. Using caregiver-reported developmental indices complemented by routine clinical assessments, the study will generate population-level comparative data on infant wellbeing during the follow-up period. These findings will contribute essential early safety signals and support national and global guidance on the use of long-acting PrEP in breastfeeding populations.

## Limitations

This study has several limitations. Enrolment from birth to five months spans a period of rapid ontogeny of Uridine Diphosphate Glucuronosyltransferase 1A1 (UGT1A1)-mediated glucuronidation and developmental change. Although population pharmacokinetic modelling incorporating maturation functions and age-standardised growth and developmental outcomes will be used, the small number of infants within age strata limits precision in characterising exposure–age relationships; findings at younger ages should therefore be interpreted with caution. Secondly, the study is not powered to detect between-group differences in infant safety, growth, or developmental outcomes, and these analyses are consequently descriptive and hypothesis-generating, with formal inference deferred to larger studies. Thirdly, infant feeding patterns and maternal nutritional status may influence infant exposure; feeding will be captured longitudinally and incorporated as a time-varying covariate, while maternal weight will be evaluated in relation to cabotegravir clearance and milk-to-plasma ratios. However, the study does not directly measure milk composition or detailed maternal dietary intake, limiting the ability to fully assess indirect dietary effects. Finally, as exposure groups are self-selected, comparative analyses are observational and exploratory, and although adjusted and sensitivity analyses will be undertaken where feasible, residual confounding cannot be excluded.

## Conclusion

Lactating women represent a key population with heightened vulnerability to HIV acquisition, and consequently, a risk of transmission to their infants. However, they remain largely underrepresented in clinical trials of novel HIV prevention agents, including long-acting cabotegravir. The CABMILK study will fill this critical evidence gap by generating foundational data on maternal, breast milk, and infant cabotegravir exposure alongside comparative assessments of infant growth and development. Findings from this study will provide early, context-specific safety data essential for informing Malawi’s national CAB-LA rollout and guiding future WHO recommendations. By strengthening the evidence base for postpartum PrEP use, this study has the potential to enhance clinical decision-making, improve community confidence in long-acting PrEP, and support equitable access to HIV prevention for lactating mothers and their infants.

## Data Availability

No participant data are associated with this article, as it describes a study protocol. Extended study materials supporting this protocol are openly available on Zenodo: https://doi.org/10.5281/zenodo.18490313 ^24^ These materials include the Statistical Analysis Plan, sample collection Case Report Forms (CRFs), and the laboratory analytical plan for cabotegravir quantification. Data are available under the terms of the Creative Commons Attribution 4.0 International (CC-BY 4.0) license. De-identified individual participant data generated from the CABMILK study, including pharmacokinetic concentration data and relevant clinical and demographic variables, will be made available after study completion and publication of the primary results. Data sharing will be conducted in accordance with participant consent, Malawi national research regulations, and institutional data governance policies. Requests for access to study data will be reviewed by the study investigators and an appropriate data access committee. Data will be shared in a de-identified format, and requestors may be required to sign a data use agreement.
