# Supplementary material for: Breastmilk transfer of cabotegravir and infant safety outcomes in mothers on cabotegravir for HIV prevention in Lilongwe, Malawi: A protocol for an observational pharmacokinetic study, CABMILK Study
Source: Wellcome Open Res. 2026 Jun 9;11:137. Originally published 2026 Feb 20. [Version 2] doi: 10.12688/wellcomeopenres.25966.2 (PMC13291744; doi:10.12688/wellcomeopenres.25966.2)
Supplement: Peer review history

# Reviewer report: Reviewer response for version 2

**DOI:** 10.21956/wellcomeopenres.29296.r157727

**Published:** 2026-06-27

**Authors:** Lisa Abuogi

**Affiliations:** 1 University of Colorado, Denver, Colorado, USA

I have reviewed the revised protocol manuscript. The authors have responded to all concerns raised on initial review.

Is the study design appropriate for the research question?

Partly

Is the rationale for, and objectives of, the study clearly described?

Yes

Are sufficient details of the methods provided to allow replication by others?

Yes

Are the datasets clearly presented in a useable and accessible format?

Not applicable

Reviewer Expertise:

Perinatal and pediatric HIV.

I confirm that I have read this submission and believe that I have an appropriate level of expertise to confirm that it is of an acceptable scientific standard.

# Reviewer report: Reviewer response for version 2

**DOI:** 10.21956/wellcomeopenres.29296.r157945

**Published:** 2026-06-23

**Authors:** Stephen Ian Walimbwa

**Affiliations:** 1 University Hospital Olomouc, Olomouc, Czech Republic

The study addresses an important knowledge gap in clinical practice as long-acting antiretrovirals become readily available. The protocol is well written, and I have only minor comments.

- Indicate within the abstract the HIV serostatus of the mother-infant pairs enrolled in the study.
- The introduction should provide more information on whether the ART class is associated with any adverse events in mother-infant pairs.
- Will renal function tests be performed for mothers on TDF-3TC?
- Keep the wording in Fig2 and elsewhere in the protocol for PK sampling relative to either CAB 5 ^th^ dose or at month-7 postpartum and not both.
- The sentence “CAB would transfer” on page 7 seems incomplete.
- Specify the EDTA tube (K2 or K3) for blood collections.

Is the study design appropriate for the research question?

Yes

Is the rationale for, and objectives of, the study clearly described?

Partly

Are sufficient details of the methods provided to allow replication by others?

Yes

Are the datasets clearly presented in a useable and accessible format?

Yes

Reviewer Expertise:

HIV, Clinical Pharmacology, Pharmacokinetics, Immunology, Infectious diseases, Vaccinology

I confirm that I have read this submission and believe that I have an appropriate level of expertise to confirm that it is of an acceptable scientific standard.

# Reviewer report: Reviewer response for version 1

**DOI:** 10.21956/wellcomeopenres.28578.r150189

**Published:** 2026-03-18

**Authors:** Stefano Rusconi

**Affiliations:** 1 Universita' degli Studi di Milano, Milan, Italy

The protocol deals with a peculiar research question. The PrEP with CAB is going through a huge implementation process, thus it's pivotal to investigate its impact in women. In particular, this issue in BF mothers is of particular interect and importance.

 The protocol is well designed, with the correct methods and time-points.

 I do not have any extra suggestions.

Is the study design appropriate for the research question?

Yes

Is the rationale for, and objectives of, the study clearly described?

Yes

Are sufficient details of the methods provided to allow replication by others?

Yes

Are the datasets clearly presented in a useable and accessible format?

Yes

Reviewer Expertise:

Infectious diseases, clinical virology, LMIC, in vitro evaluation of antivirals, bioethics.

I confirm that I have read this submission and believe that I have an appropriate level of expertise to confirm that it is of an acceptable scientific standard.

# Reviewer report: Reviewer response for version 1

**DOI:** 10.21956/wellcomeopenres.28578.r149217

**Published:** 2026-03-14

**Authors:** Lisa Abuogi

**Affiliations:** 1 University of Colorado, Denver, Colorado, USA

Wellcome Open Research

 11 ^th^ March 2026

**Breastmilk transfer of cabotegravir and infant safety outcomes in mothers on cabotegravir for HIV prevention in Lilongwe, Malawi: A protocol for an observational pharmacokinetic study, CABMILK Study**

 Summary: This manuscript describes a protocol to leverage two ongoing large-scale implementation trials of cabotegravir for HIV prevention in pregnant and breastfeeding women in Malawi to determine breastmilk exposure of cabotegravir and assess infant safety outcomes. The proposed research will address an important knowledge gap regarding the use of long-acting injectable cabotegravir for HIV prevention in this population. The proposed study is highly feasible given its ability to recruit from two large ongoing studies in Malawi to implement CAB as PrEP for pregnant or lactating women. The procedures and analytic approach to assessing breastmilk concentrations and infant exposure to CAB are rigorous. Several concerns, however, are identified. Most significantly, the study will not have a sufficient sample size to determine associations between CAB exposure and infant safety outcomes. Only 25 infants will have potential CAB exposure through breastmilk compared to 25 whose mothers are on oral PrEP and 25 on nothing. There are no power estimates to show that any of the proposed safety outcomes would be identified at a rate to show a difference between these very small numbers of infants. Further, mother-infant pairs will be enrolled at different starting time points and thus outcomes, including CAB PK and safety outcomes, may be determined at different ages for infants. There is limited discussion as to how this will be accounted for, other than mention of including infant age as one of many potential confounders that will be considered. Overall, this is an important study that would benefit from focusing on breastmilk transfer of CAB and documentation (without attempts to determine association unless it can demonstrate sufficient sample size) of infant safety outcomes. Recommended revisions are detailed below.

 Major issues:

1. Infant age will differ at sampling/study time points as mother-infant pairs will be enrolled any time from birth to 5 months of age at the time of initiation of CAB. Thus, all samples, growth and development assessments will happen at different ages, which is not well described or considered. Since infant drug metabolism changes quickly over time (as do growth and development) and the study has a small sample size, investigators need to consider this or describe it as a significant limitation.
2. Small sample sizes are generally utilized in PK studies, which is appropriate for determining CAB breastmilk exposure and relative infant dosing. However, the proposed sample sizes are not justified for evaluating an association between CAB BM exposure and safety, growth, or development outcomes. There are no power calculations or effect estimates provided that demonstrate a significant association could be detected. Rather, it may be more appropriate to simply document safety outcomes in a preliminary manner and utilize the larger implementation studies to rigorously assess these outcomes across groups.
3. Recent implementation studies, including in Malawi, have shown depressingly low continuation on injectable PrEP. Given that investigators will enroll at PrEP initiation and wait until 5 injections have been given to achieve steady state, the authors need to describe how inevitable losses to follow up or missed doses are going to be handled to achieve 25 evaluable mother-infant pairs.
4. Infant diet is noted as a potential confounder to CAB-breastmilk exposure. While infant feeding patterns will be documented, how this will be analyzed is not clear. What about the maternal diet’s impact on CAB breastmilk levels?
5. P10 Authors introduce a previously undescribed plan to develop a pilot mother-infant registry to detect rate safety outcomes. It is unclear if this is an objective of the proposed study or the larger parent studies. No further details on how such a registry would be developed, how data would be stored, who would contribute data, and how it would be managed or evaluated. Unclear if women consent to long-term data storage in a registry.
6. Analysis of infant safety outcomes is described, including adjusted regression models and consideration of several variables. Given the very small sample size, it is not clear that a regression model and confounder analysis will be possible. The authors acknowledge this, but it is not clear it should be done at all without additional justification.
7. Community engagement as described is limited to information sharing to community about the proposed study. Unclear if what is described is for the CABMILK study or the parent studies. No description of how community input will be integrated and used during the conduct of the research.

Minor issues:

  

1. No description of exclusion of premature infants whose metabolism and safety outcomes would be quite different than term infants. “Infant comorbidities” is mentioned generically without detail.
2. Describe ethical body that is reviewing the study.
3. P4. Typo … whose mothers receive oral PrEP (TDF/FTC) or no PrEP. association between breastmilk transfer.

Is the study design appropriate for the research question?

Partly

Is the rationale for, and objectives of, the study clearly described?

Yes

Are sufficient details of the methods provided to allow replication by others?

Yes

Are the datasets clearly presented in a useable and accessible format?

Not applicable

Reviewer Expertise:

Perinatal and pediatric HIV.

I confirm that I have read this submission and believe that I have an appropriate level of expertise to confirm that it is of an acceptable scientific standard, however I have significant reservations, as outlined above.

# Response

**Published:** 2026-05-21

**Authors:** Mphatso Kantonya

**Affiliations:** Infectious Diseases Epidemiology, Malawi Liverpool Wellcome Programmme, Lilongwe, Central, Malawi

**CABMILK Study – Responses to reviewer** *Breastmilk transfer of cabotegravir and infant safety outcomes in mothers on cabotegravir for HIV prevention in Lilongwe, Malawi (CABMILK Study) – Wellcome Open Research* Dear Editor and Reviewer, We thank the reviewer for a careful and constructive review of the CABMILK Study protocol. The comments have helped us to sharpen the framing of the study as a pharmacokinetic and preliminary safety-documentation exercise; to be transparent about what the study can and cannot deliver; and to make explicit the operational, developmental-pharmacology, and programmatic assumptions that underpin the design. In particular, we have adopted the reviewer’s central recommendation that the study should be positioned primarily as a breastmilk and infant pharmacokinetic study with preliminary, descriptive documentation of infant safety outcomes. We have therefore revised the objectives, analytic framework, and limitations accordingly. Below we respond to each comment in turn. Reviewer comments are reproduced verbatim in a *shaded block.* Our response follows. For each comment that resulted in a protocol revision, we additionally reproduce the actual revised text from the protocol verbatim, so that the editor and reviewer can appreciate the change without needing to consult the full protocol. Major issues ***Reviewer comment:*** *Infant age will differ at sampling/study time points as mother–infant pairs will be enrolled any time from birth to 5 months of age at the time of initiation of CAB. Thus, all samples, growth and development assessments will happen at different ages, which is not well described or considered. Since infant drug metabolism changes quickly over time (as do growth and development) and the study has a small sample size, investigators need to consider this or describe it as a significant limitation.* **Response:** We thank the reviewer for this important observation. Variability in infant age at enrolment and sampling is a key methodological consideration given rapid postnatal maturation and developmental change. We have revised the methods (lines 426 to 448) and limitations (lines 636 to 641) sections to address this explicitly. Infant age will be captured to the day at every sampling occasion and at every safety and developmental assessment, and both postnatal and postmenstrual age will be entered as time-varying variables in the population PK model. Maturation of clearance is incorporated structurally using a sigmoidal UGT1A1 ontogeny function with published parameters and tested in a pre-specified sensitivity analysis using an alternative published parameter set. Growth and developmental outcomes are age-standardised. The limitations section now explicitly acknowledges that the small number of infants within age strata limits precision in characterising exposure–age relationships, and that findings at younger ages should be interpreted with caution. ***Revised protocol text (verbatim):*** *“Data will be analysed using either STATA or R. Infant age at every sampling occasion and at every safety and developmental assessment will be recorded to the day. Both postnatal age (PNA) and postmenstrual age (PMA = gestational age at birth + PNA) will be derived for each timepoint and treated as time-varying variables.The population pharmacokinetic model (NONMEM/Monolix) will incorporate, as fixed structural features, allometric size scaling (exponent 0.75 on apparent clearance, 1 on volume of distribution) and a sigmoidal maturation function on apparent clearance, with PMA50 and the Hill coefficient set to published values for UGT1A1 ontogeny rather than estimated from this small cohort. Residual covariate effects of PNA, weight, sex, and feeding pattern will then be tested empirically. To probe the robustness of the maturation assumption, we will conduct a pre-specified sensitivity analysis re-fitting the model with at least one alternative published UGT1A1 ontogeny parameter set and report the resulting impact on individual exposure estimates.”* *“Limitations: Enrolment from birth to five months spans a period of rapid ontogeny of UGT1A1-mediated glucuronidation and developmental change. Although population pharmacokinetic modelling incorporating maturation functions and age-standardised growth and developmental outcomes will be used, the small number of infants within age strata limits precision in characterising exposure–age relationships; findings at younger ages should therefore be interpreted with caution.”* ***Reviewer comment:*** *Small sample sizes are generally utilized in PK studies, which is appropriate for determining CAB breastmilk exposure and relative infant dosing. However, the proposed sample sizes are not justified for evaluating an association between CAB BM exposure and safety, growth, or development outcomes. There are no power calculations or effect estimates provided that demonstrate a significant association could be detected. Rather, it may be more appropriate to simply document safety outcomes in a preliminary manner and utilize the larger implementation studies to rigorously assess these outcomes across groups.* **Response:** We fully agree with the reviewer that the study is not powered to detect statistically significant associations between cabotegravir exposure and infant safety outcomes. The primary aim of CABMILK is pharmacokinetic characterisation, and the safety component is intended to provide preliminary, hypothesis-generating data rather than definitive comparative inference. We have therefore reframed the objectives (lines 60 to 65, 135 to 139, and 145 to 149), the Step 2 description (lines 398 to 409), and the analysis plan to remove inferential language and to position safety, growth, and developmental outcomes as descriptive characterisations with effect sizes and 95% confidence intervals. Powered comparative safety evaluation is deferred to the larger PathToScale and LINK parent studies and to regional pharmacovigilance. On the related question of attrition: because the primary PK analysis depends on completing maternal plasma, breastmilk and infant samples at the month-7 (pre-fifth-injection) visit, any participant who discontinues CAB-LA or is lost to follow-up before that point would not contribute to the primary PK analysis. The protocol therefore specifies that recruitment will continue until 25 evaluable mother–infant pairs have been achieved in the CAB-LA group. In effect, mothers who drop out before the month-7 PK visit are replaced by additional enrolment, so that the study remains powered for the primary PK objective. Mothers who drop out after providing the month-7 PK samples are not replaced because their PK data are already informative; their loss is documented descriptively as part of the longitudinal safety follow-up. Baseline characteristics of mothers who do and do not reach the evaluable PK sampling visit will be compared descriptively to allow the reader to assess the representativeness of the evaluable cohort. ***Revised protocol text (verbatim):*** *“Step 2 — Cabotegravir safety, growth pattern and development study: Step 2 is designed to document the pattern of adverse events, growth trajectories, and developmental scores observed in CAB-LA-exposed breastfed infants over eight months of follow-up, with contextual description of the same outcomes in infants whose mothers are receiving oral PrEP or no PrEP. This step is not powered to detect between-group differences; any apparent differences will be reported with 95% confidence intervals and interpreted only as signals to be formally evaluated in the larger parent implementation studies (PathToScale, LINK) and in regional pharmacovigilance.”* *“Retention and evaluable pairs: The primary pharmacokinetic analysis will be conducted on mother–infant pairs who contribute matched maternal plasma, breastmilk, and at least one infant dried blood spot sample within the month-7 pre-fifth-injection window. Losses before the month-7 sampling visit, missed CAB-LA doses, and out-of-window sampling will be documented and reported as implementation outcomes. Baseline characteristics of mothers who do and do not reach the evaluable PK sampling visit will be compared descriptively. Retention is supported through appointment reminders, alignment of CABMILK visits with routine postnatal and under-five clinic contacts, travel reimbursement, and, where acceptable to the mother, a home visit to complete a missed sampling occasion. Recruitment will continue until the target of 25 evaluable pairs in the CAB-LA group has been met.”* *“Data analysis: Primary safety results will be descriptive: counts, proportions with exact 95% confidence intervals, incidence rates, and Kaplan–Meier curves, without group-comparison p-values. Longitudinal growth and CREDI outcomes will be analysed with mixed-effects models adjusting for a minimal pre-specified set of covariates: infant age at measurement, infant sex, and site. Formal between-group inference for safety is deferred to other larger PK studies.”* ***Reviewer comment:*** *Recent implementation studies, including in Malawi, have shown depressingly low continuation on injectable PrEP. Given that investigators will enrol at PrEP initiation and wait until 5 injections have been given to achieve steady state, the authors need to describe how inevitable losses to follow up or missed doses are going to be handled to achieve 25 evaluable mother–infant pairs.* **Response:** We thank the reviewer for highlighting this. We acknowledge that retention on CAB-LA may be suboptimal in real-world settings. The protocol now defines the evaluable cohort and the strategy for achieving 25 evaluable mother–infant pairs explicitly (lines 514 to 534). The primary PK analysis will be restricted to mother–infant pairs who provide matched maternal plasma, breastmilk and infant dried blood spot samples in the month-7 (pre-fifth-injection) window. Losses, missed doses and out-of-window sampling are reported as implementation outcomes. To preserve power for the primary PK objective, recruitment will continue until 25 evaluable pairs have been achieved in the CAB-LA group, that is, mothers lost before the month-7 PK visit are replaced by additional enrolment. where this is not possible, with time constraints, the investigators will only analyse the available participant samples. Retention is actively supported through appointment reminders, alignment with routine postnatal and under-five clinic contacts, travel reimbursement, and home visits where acceptable to the mother. Baseline characteristics of mothers who reach versus do not reach the PK sampling visit will be compared descriptively. ***Revised protocol text (verbatim):*** *“Missed visits: All dosing visits for injectable and oral PrEP are aligned with those of the parent PathToScale and LINK implementation studies. As the CABMILK study is leveraging the platform of the implementation of PathToScale and LINK studies, missed dosing or follow-up visits are inevitable. Nevertheless, any such missed dosing or follow-up visits, including sample collection visits, will be conducted as soon as it is possible to do so. This will be documented appropriately in the participant’s file.”* *“Retention and evaluable pairs: The primary pharmacokinetic analysis will be conducted on mother–infant pairs who contribute matched maternal plasma, breastmilk, and at least one infant dried blood spot sample within the month-7 pre-fifth-injection window. Losses before the month-7 sampling visit, missed CAB-LA doses, and out-of-window sampling will be documented and reported as implementation outcomes. Baseline characteristics of mothers who do and do not reach the evaluable PK sampling visit will be compared descriptively. Retention is supported through appointment reminders, alignment of CABMILK visits with routine postnatal and under-five clinic contacts, travel reimbursement, and, where acceptable to the mother, a home visit to complete a missed sampling occasion. Recruitment will continue until the target of 25 evaluable pairs in the CAB-LA group has been met.”* ***Reviewer comment:*** *Infant diet is noted as a potential confounder to CAB-breastmilk exposure. While infant feeding patterns will be documented, how this will be analysed is not clear. What about the maternal diet’s impact on CAB breastmilk levels?* **Response:** We thank the reviewer for this point. Infant feeding category will be captured at every visit (exclusive breastfeeding, mixed feeding, complementary feeding, replacement feeding, and the timing and frequency of feeds relative to sampling) and entered into pharmacokinetic and safety analyses as a time-varying covariate. With respect to maternal diet, cabotegravir is highly protein-bound (>99%) and predominantly lipophilic; the intramuscular depot release that drives CAB-LA systemic exposure is not expected to be substantially influenced by a single meal or by dietary composition. Nevertheless, maternal nutritional status more broadly, captured through pre-injection weight and BMI, may influence depot release, apparent clearance, and milk composition. Maternal pre-injection weight will therefore be tested as a covariate on cabotegravir apparent clearance and on the milk-to-plasma ratio in the population PK model. We acknowledge as a limitation (lines 644 to 648) our inability to measure milk fat fraction or detailed dietary intake directly, and therefore to fully resolve any indirect effect of maternal diet on infant exposure. ***Revised protocol text (verbatim):*** *“Information on other factors that could impact CAB breastmilk exposure: Two classes of variable are recognised as potentially affecting cabotegravir concentrations in breastmilk and the resulting infant exposure: (a) infant feeding pattern, and (b) maternal nutritional status. Infant feeding will be captured at every visit using standard feeding indicators (exclusive, mixed, complementary, replacement) and the timing and frequency of feeds relative to sampling and will be entered into pharmacokinetic and safety analyses as a time-varying covariate. Maternal pre-injection weight will be measured at baseline and at each dosing visit and evaluated as covariates on cabotegravir apparent clearance and on the milk-to-plasma ratio in the population PK model. We acknowledge as a limitation that the study does not directly measure milk fat fraction or detailed maternal dietary intake, and therefore cannot fully resolve any indirect effect of maternal diet on infant cabotegravir exposure.”* ***Reviewer comment:*** *P10 Authors introduce a previously undescribed plan to develop a pilot mother–infant registry to detect rate safety outcomes. It is unclear if this is an objective of the proposed study or the larger parent studies. No further details on how such a registry would be developed, how data would be stored, who would contribute data, and how it would be managed or evaluated. Unclear if women consent to long-term data storage in a registry.* **Response:** We thank the reviewer for identifying this lack of clarity. The reference to a “pilot registry” was intended to describe the structured longitudinal follow-up of mother–infant pairs within this study rather than a separate long-term registry infrastructure. To avoid confusion, we have removed the term “registry” throughout the protocol and replaced it with “prospective observational cohort follow-up” (particularly lines 398 to 404). All data collection, storage, and management occur within the CABMILK study framework under the existing ethical approvals, informed consent, and MLW data-governance procedures, with samples and data retained for a maximum of five years after study completion and publication. Any longer-term observational cohort of CAB-LA-exposed mother–infant pairs would be pursued under a separate protocol, separate ethical approval, and re-consent of participants. ***Revised protocol text (verbatim):*** *“Step 2 (Prospective observational cohort follow-up of mother–infant pairs): Detecting rare safety outcomes or subtle neurodevelopmental effects related to cabotegravir exposure would require substantially larger and longer-term studies. Accordingly, Step 2 is designed as a prospective observational cohort to describe infant exposure and characterise safety signals rather than to support formal inferential comparisons.”* *“All data collection, storage, and management within both steps occur inside the CABMILK study framework under existing ethical approvals, informed consent, and MLW data-governance procedures. Samples and data are retained for a maximum of five years after study completion and publication. Any future longer-term observational cohort of CAB-LA-exposed mother–infant pairs would require a separate protocol, separate ethical approval, and re-consent of participants.”* ***Reviewer comment:*** *Analysis of infant safety outcomes is described, including adjusted regression models and consideration of several variables. Given the very small sample size, it is not clear that a regression model and confounder analysis will be possible. The authors acknowledge this, but it is not clear it should be done at all without additional justification.* **Response:** We agree that the use of multivariable regression for infant safety outcomes is limited by the small sample size. The analysis plan has been revised to prioritise descriptive analyses (counts and proportions with exact 95% confidence intervals, incidence rates, and Kaplan–Meier curves) and to restrict regression models to the longitudinal growth and CREDI endpoints, where the unit of analysis is the repeated measurement rather than the infant. For these, a minimal pre-specified covariate set (infant age at measurement, infant sex, and site) is used to avoid overfitting. Regression findings are interpreted as exploratory effect-size estimates rather than as formal hypothesis tests, and formal between-group safety inference is deferred to the larger studies. ***Revised protocol text (verbatim):*** *“Primary safety results will be descriptive: counts, proportions with exact 95% confidence intervals, incidence rates, and Kaplan–Meier curves, without group-comparison p-values. Longitudinal growth and CREDI outcomes will be analysed with mixed-effects models adjusting for a minimal pre-specified set of covariates: infant age at measurement, infant sex, and site. Formal between-group inference for safety is deferred to other larger PK studies. Where feasible, we will perform sensitivity analyses to reduce imbalance, recognising limitations due to our sample size.”* ***Reviewer comment:*** *Community engagement as described is limited to information sharing to community about the proposed study. Unclear if what is described is for the CABMILK study or the parent studies. No description of how community input will be integrated and used during the conduct of the research.* **Response:** We appreciate this important observation. While community members were not directly involved in the initial planning phase of the study, we have conducted community engagement activities during study set-up and are continuing them throughout implementation. These include sensitisation meetings with hospital staff, community leaders, and ongoing dialogue with mothers and caregivers in Chichewa at the under-five and postnatal clinics. Insights from these interactions are reviewed by the study team and used to refine study procedures, communication materials, and the participant experience. We have clarified in the manuscript (lines 558 to 567) that these activities are specific to CABMILK and are co-delivered with, but distinct from, the community-engagement architecture of the parent PathToScale and LINK studies. ***Revised protocol text (verbatim):*** *“Community engagement in CABMILK will be initiated at study set-up and continue throughout implementation. Sensitisation meetings will be held with hospital staff, and later with community leaders, and residents in the catchment areas of the participating health centres. Ongoing engagement will take place through listening sessions with enrolled mothers and caregivers in Chichewa at the under-five and postnatal clinics, and through community-engagement activities broadly organised by the institution’s Community Engagement Department aimed at increasing visibility in Lilongwe, where its research activities are not yet well established. From these activities, we aim to enhance enrolment and retention in the CABMILK study. These CABMILK-specific activities are distinct from, but co-delivered with, the broader community-engagement architecture of the parent PathToScale and LINK studies. These activities ensure that the study remains transparent, culturally grounded, and responsive to community needs.”* Minor issues ***Reviewer comment:*** *No description of exclusion of premature infants whose metabolism and safety outcomes would be quite different than term infants. “Infant comorbidities” is mentioned generically without detail.*

**Response:** We thank the reviewer for this important point. We have chosen not to exclude preterm infants categorically, in order to preserve the real-world, programmatic character of the study; breastfeeding women receiving CAB-LA under the PathToScale and LINK programmes include those whose infants were born preterm, and their exclusion would reduce generalisability. Enrolment is, however, restricted to infants with a birthweight ≥1.5 kg, which excludes extremely preterm and clinically unstable neonates in whom PK, safety, and outcome assessment would not be interpretable. Cabotegravir is predominantly cleared by hepatic UGT1A1-mediated glucuronidation, which matures rapidly in the first months of life; because pharmacokinetic sampling occurs after four CAB-LA maternal injections (around seven months post-enrolment), most infants – including those born preterm – will have substantially matured metabolic capacity by the time of the primary PK assessment. Gestational age at birth will be recorded at enrolment and used as a covariate in analyses; residual variability attributable to prematurity is acknowledged as a limitation. We have additionally specified the categories of infant comorbidity that constitute exclusion criteria. The relevant section of the methodology has been revised (lines 222 to 226).

***Revised protocol text (verbatim):*** *“Mother–infant dyads will be excluded if either the mother or infant is receiving concomitant medications known to significantly alter cabotegravir exposure, including rifamycin-based tuberculosis prevention or treatment. Enrolment will be restricted to infants with a birthweight ≥1.5 kg; extremely preterm and clinically unstable neonates are therefore excluded, but otherwise well preterm infants are eligible in order to preserve generalisability to the real-world population receiving CAB-LA under the parent studies. Gestational age at birth will be recorded at enrolment and used as a covariate in analyses. Dyads will also be excluded if the infant has a documented congenital anomaly expected to affect drug disposition or survival. Severe anaemia, defined as haemoglobin concentration <5 g/dL, will constitute an exclusion criterion.”*

***Reviewer comment:*** *Describe ethical body that is reviewing the study.*

**Response:** We thank the reviewer. We have clarified the ethical oversight arrangements in the Ethical Considerations section (lines 453 to 460).

***Revised protocol text (verbatim):*** *“The CABMILK study protocol, participant information sheet, and informed consent documents have been reviewed and approved by the National Health Sciences Research Committee (NHSRC) of Malawi (approval reference 24/10/4527), with institutional ethical oversight from the Malawi-Liverpool-Wellcome Research Programme Clinical Research Support Unit (CRSU). The study will be conducted in accordance with the Declaration of Helsinki (2013) and ICH Good Clinical Practice (E6 R2, and R3 as applicable). Any protocol amendments will be submitted to the NHSRC before implementation, and serious adverse events will be reported within the regulatory timelines.”*******

***Reviewer comment:*** *P4. Typo … whose mothers receive oral PrEP (TDF/FTC) or no PrEP. association between breastmilk transfer.*

**Response:** Thank you, this typo has been corrected on page 4 as follows: *“…to characterise cabotegravir transfer into breastmilk and the resulting infant exposure among breastfeeding women receiving long-acting injectable cabotegravir for HIV pre-exposure prophylaxis, and to describe, in a preliminary, non-inferential manner, the infant safety, growth, and developmental patterns in this group relative to infants whose mothers receive oral PrEP (TDF/FTC) or no PrEP.”* We thank the reviewer again for comments that have substantially strengthened the clarity, statistical honesty, and operational feasibility of the protocol. The revised protocol now aligns with the reviewer’s recommendation that CABMILK focus on what it is best positioned to deliver: a rigorous characterization of cabotegravir transfer into breastmilk and infant exposure, while transparently documenting safety outcomes and deferring adequately-powered safety inference to the larger parent studies and to regional pharmacovigilance. We look forward to the editor’s decision.

**On behalf of all co-authors,** Mphatso S. Kantonya, *Corresponding author – CABMILK Study* Malawi-Liverpool-Wellcome Research Programme, Blantyre, Malawi mkantonya@mlw.mw
